# Assessing health status over time: impact of recall period and anchor question on the minimal clinically important difference of copd health status tools

**DOI:** 10.1186/s12955-018-0950-7

**Published:** 2018-06-26

**Authors:** H. J. Alma, C. de Jong, D. Jelusic, M. Wittmann, M. Schuler, B. J. Kollen, R. Sanderman, K. Schultz, J. W. H. Kocks, T. Van der Molen

**Affiliations:** 1 University of Groningen, University Medical Center Groningen, Department of General Practice and Elderly Care Medicine, HPC FA21, Postbox 196, NL-9700 AD Groningen, The Netherlands; 2 University of Groningen, University Medical Center Groningen, Groningen Research Institute for Asthma and COPD (GRIAC), Groningen, The Netherlands; 3 Klinik Bad Reichenhall, Center for Rehabilitation, Pulmonology and Orthopedics, Bad Reichenhall, Germany; 4 University of Wuerzburg, Department of Medical Psychology and Psychotherapy, Medical Sociology and Rehabilitation Sciences, Wuerzburg, Germany; 5 University of Groningen, University Medical Center Groningen, Department of Health Psychology, Groningen, The Netherlands; 6 University of Twente, Department of Psychology, Health and Technology, Enschede, The Netherlands

**Keywords:** Chronic obstructive pulmonary disease (COPD), Health status, Clinical COPD questionnaire (CCQ), COPD assessment test (CAT), St. George’s respiratory questionnaire (SGRQ), Minimal clinically important difference, Clinically relevant change, Global rating of change scale, Recall period, Pulmonary rehabilitation

## Abstract

**Background:**

The Minimal Clinically Important Difference (MCID) assesses what change on a measurement tool can be considered minimal clinically relevant. Although the recall period can influence questionnaire scores, it is unclear if it influences the MCID. This study is the first to examine longitudinally the impact of the recall period of an anchor question and its design on the MCID of COPD health status tools using the COPD Assessment Test (CAT), Clinical COPD Questionnaire (CCQ) and the St. George’s Respiratory Questionnaire (SGRQ).

**Methods:**

Moderate to very severe COPD patients without respiratory co-morbidities were recruited during 3-week Pulmonary Rehabilitation (PR). CAT, CCQ and SGRQ were completed at baseline, discharge, 3, 6, 9 and 12 months. A 15-point Global Rating of Change scale (GRC) was completed at each follow-up. A five-point GRC was used as second anchor at 12 months. Mean change scores of a subset of patients indicating a minimal improvement on each of the anchor questions were considered the MCID. The MCID estimates over different time periods were compared with one another by evaluating the degree of overlap of Confidence Intervals (CI) adjusted for dependency.

**Results:**

In total 451 patients were included (57.9 ± 6.6 years, 65% male, 50/39/11% GOLD II/III/IV), of which 309 completed follow-up. Baseline health status scores were 20.2 ± 7.3 (CAT), 2.9 ± 1.2 (CCQ) and 50.7 ± 17.3 (SGRQ). MCID estimates for improvement ranged − 3.1 to − 1.4 for CAT, − 0.6 to − 0.3 for CCQ, and − 10.3 to − 7.6 for SGRQ. Absolute higher – though not significant – MCIDs were observed for CAT and CCQ directly after PR. Significantly absolute lower MCID estimates were observed for CAT (difference − 1.4: CI -2.3 to − 0.5) and CCQ (difference − 0.2: CI -0.3 to −0.1) using a five-point GRC.

**Conclusions:**

The recall period of a 15-point anchor question seemed to have limited impact on the MCID for improvement of CAT, CCQ and SGRQ during PR; although a 3-week MCID estimate directly after PR might lead to absolute higher values. However, the design of the anchor question was likely to influence the MCID of CAT and CCQ.

**Trial registration:**

RIMTCORE trial #DRKS00004609 and #12107 (Ethik-Kommission der Bayerischen Landesärztekammer).

**Electronic supplementary material:**

The online version of this article (10.1186/s12955-018-0950-7) contains supplementary material, which is available to authorized users.

## Background

Health status can be defined as *“the impact of health on a person’s ability to perform and derive fulfilment from the activities of daily life”* [1]. Its measurement is a standardized means of quantifying this impact on a patient’s daily life, health and wellbeing [1, 2]. Multiple general- and disease-specific health status tools have been developed to detect and quantify health status [3, 4]. Physiological measures alone do not reflect the full impact of the disease and correlations with Health-Related Quality of Life (HRQoL) are often weak [4]. Determining treatment effects requires a parameter that assesses to what extent change on a health status tool can be considered clinically relevant. The Minimal Clinically Important Difference (MCID) is used to evaluate this. It has been defined as *“the smallest difference in score, which patients perceive as beneficial and which would mandate a change in the patient’s management”* [5]. Observed change should exceed the estimated MCID value in order to be clinically relevant.

MCID estimates can be determined using both anchor- and distribution-based methods [6–8]. A frequently applied anchor-based technique is the use of a reference (anchor) question, requiring patients to retrospectively assess their current health state compared to a prior measurement in time or their experienced degree of change over time [6–8]. This anchor question usually consists of multiple ordinal reply options varying from *much worse, a little worse, no change, a little better* up to *much better* [9, 10]. The technique may also be referred to as *patient-referencing* [6]. In the literature, several descriptions are used for this kind of anchor question: Global Rating scale of Change (GRC), Patient Global Impression of Change, Global Perceived Change, Transition Rating Scale and many more [9, 10]. The MCID of a health status instrument can be determined by calculating the mean change score observed for those patients indicating a minimal change (*little better or little worse*) on the anchor question, assuming data being normally distributed [9].

The use of these patient rating scales has pros and cons. Its main strengths are the ease of administration and MCID determination, as well as the involvement of a patient-related clinical anchor [9]. However, it remains unclear over which period of time change on a GRC should be assessed and how many answering options the anchor question should include. When assessing change over a longer period of time, it might be more difficult for the patient to recall their former health state. A longer recall period could result in a different MCID [10]. On the other hand, shorter periods of measurement may not reflect real change. There is no golden standard in defining an instrument’s MCID [11].

In COPD much focus is nowadays on health status measurement [12, 13], because spirometry assessment has only a weak to moderate correlation with the patient’s wellbeing [14, 15]. The COPD Assessment Test (CAT) [16], the Clinical COPD Questionnaire (CCQ) [17], and the St. George’s Respiratory Questionnaire (SGRQ) [18] are recommended by the Global initiative for Chronic Obstructive Lung Disease (GOLD) for the assessment of COPD in order to determine whether a patient is symptomatic and to what extent therapy has been successful [19]. CAT and CCQ are most applicable in clinical practice, and SGRQ in scientific research [19, 20].

Various studies examined the MCID of the CCQ to be 0.40–0.50 [21–26], including three studies using an anchor question with recall periods ranging from two to three days [21] up to three weeks [25] and eight weeks [23]. The MCID of the CAT was estimated to be two to three points [24–28], of which three studies used an anchor question with recall periods of three weeks [25] and eight weeks [27, 28]. For the SGRQ, the MCID of four points is frequently used in clinical trials. However, estimates in the literature range from four to eight points [25, 29–31], of which two studies used patient-referencing techniques with recall periods of three weeks [25] and sixteen weeks [29, 31]. No studies have investigated the influence of the recall period of the anchor question and the number of its ordinal reply categories upon the MCID of these instruments. Therefore, this study aimed to investigate the impact of the length of the anchor’s recall period and the number of reply options on the GRC on the MCID of the most frequently used health status tools CAT, CCQ and SGRQ in patients with COPD recruited from a Pulmonary Rehabilitation (PR) setting.

## Patients and methods

### Study subjects

The Routine Inspiratory Muscle Training within COPD Rehabilitation (RIMTCORE) study was a real-life randomized controlled trial (trial number #DRKS00004609) in the Klinik Bad Reichenhall, Center for Rehabilitation, Pulmonology and Orthopedics in Germany [32]. Patients were included between February 2013 and July 2014. Detailed inclusion- and exclusion criteria have been published elsewhere [25, 32]. This study is a secondary analysis of a subsample including COPD participants GOLD II-IV aged ≥18 years, who gave informed consent, without respiratory co-morbidities (e.g. bronchiectasis, asthma, history of bronchial carcinoma, sarcoidosis, tuberculosis), or alpha-1-antitrypsin deficiency.

### Study design and data collection

Patients participated in an intensive three-weeks full-day inpatient rehabilitation program tailored to the patient’s individual needs including components of physical training, education, smoking cessation, physiotherapy and counselling [25, 32]. Patient characteristics and post-bronchodilator spirometry were collected at baseline and after three weeks at the end of PR. Primary parameters collected for this sub-study were the CAT (no recall period), CCQ (weekly version) and SGRQ (monthly version) at baseline, discharge and during follow-up measurements at three, six, nine and twelve months. Measurements were taken in the clinic before and after PR. Patients were blinded to their previous answers during PR. The remaining follow-up questionnaires were sent to the patient’s home by regular mail.

The CAT is an eight-item one-dimensional scale with item scores ranging from zero to five (zero: no impairment; five: maximum impairment), summing up to a total of maximum 40 points [16]. The CCQ consists of ten items scoring from zero to six (zero: no impairment; six: maximum impairment) [17]. Domain scores (symptoms, functional status and mental status) and the total questionnaire score can be determined by summing all relevant item scores divided by the number of items. The SGRQ has 50 items divided over the domains symptoms, activities and impact [18]. Scores are calculated using the developers’ scoring file. Domain and total SGRQ scores can range from zero to 100 (zero: no impairment, 100: maximum impairment). Scores of CAT and CCQ were multiplied and standardized into a scale from zero to 100 to be comparable with SRGQ. All three questionnaires were validated and reliable in primary and secondary care, as well as PR for COPD patients [18, 29, 33, 34]. The tools are recommended according to the GOLD guidelines [19].

At each follow-up moment a 15-point Likert scale GRC anchor question was scored by the patient requiring assessment of their global health in relation to COPD compared with the start of PR (see Additional file 1: Figure S1). Answers were marked on a scale from − 7 to + 7, ranging from *very much worse* to *very much better* and zero equalling *no change* [9]. At 12-months follow-up a five-point GRC, analogue to the second question of the SF-36, was also scored by the patient (see Additional file 1: Figure S2) [35]. It required patients to rate their general health compared to one year prior. Patients could assess their status as *the same*, *somewhat better* or *somewhat worse*, or as *much better* or *much worse*. Both GRCs are frequently used in MCID research [9]. The term recall period in this sense, refers to the recall period of the GRCs.

### Determining the MCID

Scores for CAT, CCQ and SGRQ refer to their total scores. All change scores on the three questionnaires were calculated as the difference between baseline and each respective follow-up measurement. Negative change on these health status tools indicated improvement and positive change represented deterioration in HRQoL. Changes on these instruments were categorized using the corresponding score on the GRC anchor question. Scores of 0 and ± 1 on the 15-point GRC indicated *no change*; scores of ±2 and ± 3 represented a *minimal change*; scores of ±4 and ± 5 were summarized as a *moderate change*; and scores of ±6 and ± 7 indicated a *large change* [9]. The five-point GRC resulted in a division of patients as not changed, somewhat better, somewhat worse, much better, or much worse [35]. MCID estimates for the CAT, CCQ and SGRQ total scores were calculated as the mean change scores compared with baseline including the 95% Confidence Interval (95%CI) of those patients indicating a minimal improvement (+ 2 and + 3) on the GRC at each follow-up measurement, after checking for normality of distribution of the data. In addition to the 15-point Likert GRC scale, the five-point anchor question was used in a similar way to classify patients as *somewhat better*. Only patients that indicated an improvement on the GRC were included, since patients tend to get better after intervention and a limited number of patients were expected to deteriorate.

### Data analysis

Data analysis was performed using SPSS 23.0 (IBM, Chicago, USA). Descriptive data were evaluated at baseline for either frequencies with percentages (%), mean with Standard Deviation (SD) or median with range. This was depending on the variable characteristics and/or normality of distribution. CAT, CCQ and SGRQ were evaluated at baseline (T0), at discharge (T1), after three months (T2), after six months (T3), after nine months (T4) and after twelve months (T5). Normality of distribution was assessed using histograms combined with skewness and kurtosis results. Values between − 1 and + 1 were considered indicative for normality. Mean and standard deviations (or median and range) were calculated for each measurement. Data were checked for floor- and ceiling effects defined as more than 15% of the patients in the lowest and highest 10% of the maximum scale score [36]. All health status change scores were calculated between baseline and each follow-up measurement. These change scores were tested for significance using paired t-tests after verifying normality of distribution. All tests were assessed for significance using the level *p* < 0.05.

The MCID determination process included several steps. First, correlations between the GRC anchor questions, and the CAT, CCQ or SGRQ were assessed using Pearson or Spearman correlation coefficients depending on normality of distribution. Correlations needed to be ≥0.30 (preferably ≥0.50) to be eligible as anchor [7]. Next, participants were categorized according to their GRC score at each follow-up measurement. The respective change versus baseline was tested for significance using paired t-tests after checking for normality. Each MCID estimate was calculated as the mean change score compared with baseline including its 95% CI for those patients indicating a minimal improvement/somewhat better on the GRC for each follow-up moment. Correspondence between the 15-point and five-point GRC was analysed using cross tabulations, correlation coefficients and bar charts.

All MCID estimates were tested for significance with one another by determining the degree of overlap of the adjusted CIs. Due to the dependency of the data, the Intra Class Correlation Coefficient (ICC) between follow-up measurement and baseline was calculated and used to construct CIs. Adjusted CIs were calculated based on the ICC between follow-up moment and baseline [37]. The degree of dependency affects the width of the CI required to be able to test for significant differences between the various MCID estimates. Results were visualized in plots. A lack of overlap between the MCID estimates and their respective CI indicated significant differences between MCIDs. Finally, the MCID estimates and their adjusted CIs from the current study were also compared with the available thresholds from the literature (CAT 2.00, CCQ 0.40, and SGRQ 4.00 points).

## Results

### Patient characteristics

**Fig. 1:**
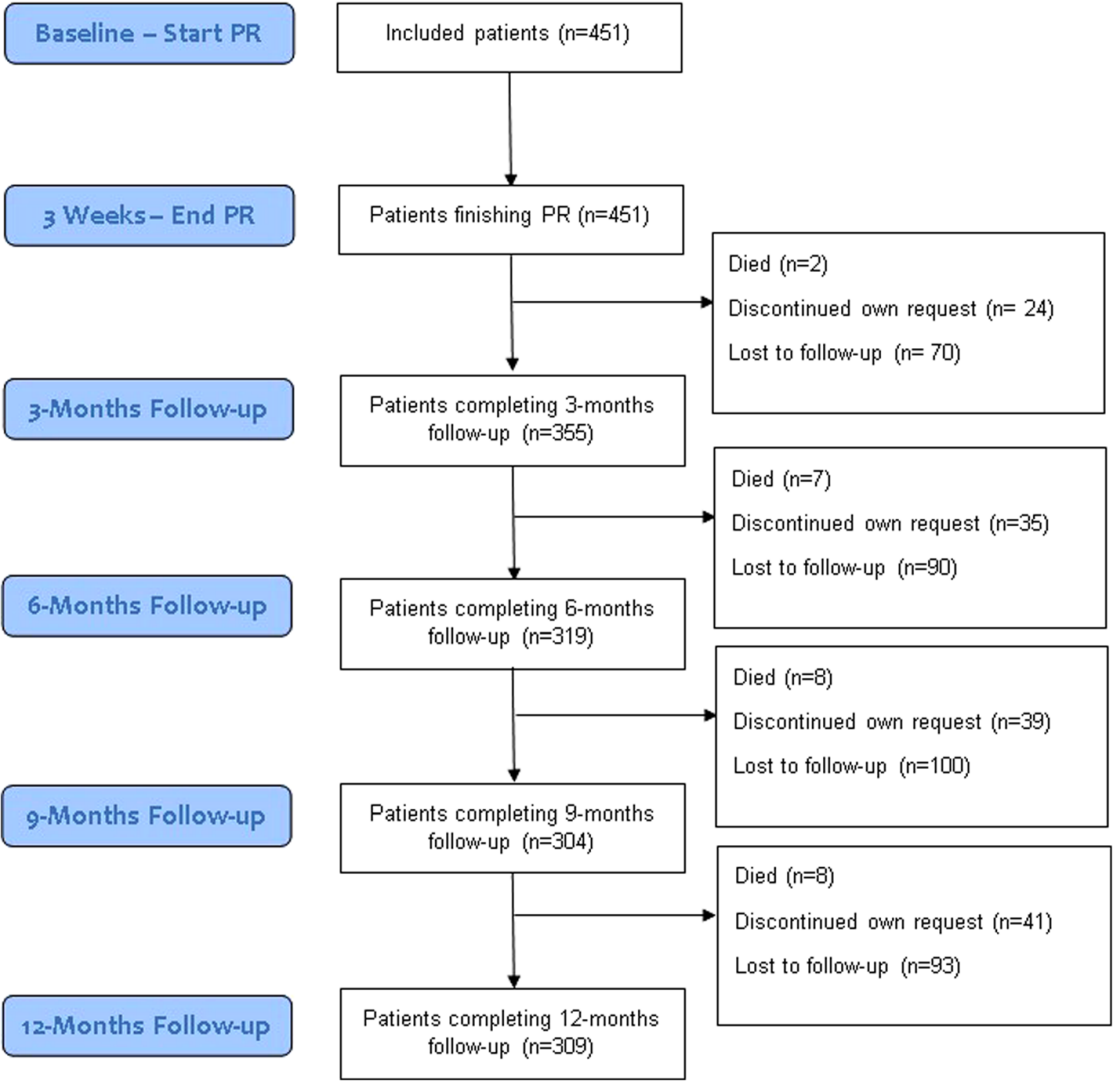
Consort flow-chart of the number of patients during follow-up

This secondary analysis of the RIMTCORE trial included 451 patients [32]. All patients had completed baseline data and at discharge, with the exception for one incomplete CCQ questionnaire, two incomplete CAT questionnaires and four incomplete SGRQ questionnaires at discharge. During follow-up 355 patients had completed data after three months; 319 after six months; 304 after nine months; and 309 after twelve months (Fig. 1). In total, eight patients died during follow-up according to our knowledge, 41 dropped out at own request and a varying number of non-responses at follow-up was present. Mean age was 58 years, 65% was male and had a mean Forced Expiratory Volume in 1 s % predicted (FEV1%pred) of 50.4 ± 15.1 (Table 1). There were no significant baseline differences between patients completing the 12-months follow-up and those who did not. Full patient characteristics at baseline have been published elsewhere [25].

**Table 1:** Baseline patient characteristics

	Baseline all patients *N* = 451	Patients with complete follow-up *N* = 309	Patients with incomplete follow-up *N* = 142	Significance testing
Age (years)^a^	57.9 ± 6.6	58.1 ± 6.5	57.5 ± 6.6	*p* = 0.39
Gender (male)^b^	65.0	63.8	67.6	*p* = 0.43
FEV_1_%pred^a^	50.4 ± 15.1	50.6 ± 14.9	50.0 ± 15.6	*p* = 0.72
GOLD II^b^	227 (50.3)	158 (51.1)	69 (48.6)	
GOLD III^b^	176 (39.0)	121 (39.2)	55 (38.7)	*p* = 0.63
GOLD IV^b^	48 (10.6)	30 (9.7)	18 (12.7)	
Smoking pack years ^a^	42.6 ± 23.5	41.1 ± 23.1	45.9 ± 24.0	*p* = 0.05
CAT ^a^	20.2 ± 7.3	20.0 ± 7.6	20.8 ± 6.8	*p* = 0.28
CCQ Total^a^	2.9 ± 1.2	2.8 ± 1.2	3.0 ± 1.1	*p* = 0.06
SGRQ Total^a^	50.7 ± 17.3	49.6 ± 17.8	53.0 ± 16.2	p = 0.05

*CAT* COPD Assessment Test, *CCQ* Clinical COPD Questionnaire, *FEV1%pred* Forced Expiratory Volume in 1 Second % predicted, *GOLD* Global initiative for Chronic Obstructive Lung Disease, *N* Number of Patients, *SGRQ* St. George’s Respiratory Questionnaire

^a^Data expressed as mean ± SD

^b^Data expressed as frequencies (% of total)

Significance tested with independent t-tests or Chi Square tests at level *p* < 0.05

### Health status scores

CAT, CCQ and SGRQ total scores were normally distributed for all measurement moments between T0 and T5. Completed pairs of change scores (follow-up vs. baseline) were included only (pair-wise deletion). There were no floor- and ceiling effects observed. There were no significant baseline differences in health status between complete and incomplete follow-up patients (Table 1). Mean baseline scores were 20.2 ± 7.3 (CAT), 2.9 ± 1.2 (CCQ) and 50.7 ± 17.3 (SGRQ) (Table 1). Mean change after twelve months follow-up was significant compared with baseline of -0.9 (95% CI -1.7 to -0.1) for CAT; -0.2 (95% CI -0.3 to -0.1) for CCQ; and -3.9 (95% CI -5.7 to -2.2) for SGRQ (Table 2).

**Table 2:** Health status baseline and change scores

	N	CAT	CAT Standardized	CCQ Total	CCQ Total Standardized	SGRQ Total	
Change at discharge (T1)	451	− 3.1* (− 3.6 to − 2.6)	− 7.8* (− 9.1 to − 6.5)	−0.6* (− 0.7 to − 0.5)	− 9.7* (− 11.2 to − 8.3)	− 9.0* (− 10.2 to − 7.9)	
Change after 3 months (T2)	355	− 1.4* (− 2.2 to − 0.7)	−3.6* (− 5.4 to − 1.8)	− 0.3* (− 0.4 to − 0.2)	− 4.3* (− 6.2 to − 2.5)	− 5.4* (− 6.9 to − 3.8)	
Change after 6 months (T3)	319	−0.9* (− 1.7 to − 0.1)	−2.3* (− 4.2 to − 0.4)	− 0.1 (− 0.2 to zero)	−1.8 (− 3.8 to + 0.2)	− 4.9* (− 6.5 to − 3.2)	
Change after 9 months (T4)	304	−1.1* (− 1.9 to − 0.4)	−2.9* (− 4.8 to − 0.9)	− 0.2* (− 0.4 to − 0.1)	− 3.8* (− 5.8 to − 1.8)	− 5.2* (− 6.9 to − 3.4)	
Change after 12 months (T5)	309	−0.9* (− 1.7 to − 0.1)	− 2.2* (− 4.2 to − 0.3)	− 0.2* (− 0.3 to − 0.1)	− 2.7* (− 4.7 to − 0.7)	−3.9* (− 5.7 to − 2.2)	

Change scores reported as mean (95%CI). Change scores were calculated in comparison to baseline. Negative values represent improvement for CAT, CCQ and SGRQ. CAT and CCQ were standardized into a scale from zero to 100 to be comparable with SGRQ

*95% CI* 95% Confidence Interval, *CAT* COPD Assessment Test, *CCQ* Clinical COPD Questionnaire, *N* Number of patients, *SGRQ* St. George’s Respiratory Questionnaire, *T1* Measurement at discharge, *T2* 3 months follow-up, *T3* 6 months follow-up, *T4* 9 months follow-up, *T5* 12 months follow-up

*Significance of change scores *p* < 0.05 at T1/T2/T3/T4/T5 compared to baseline (T0)

### Minimal clinically important differences for CAT, CCQ and SGRQ

**Fig. 2:**
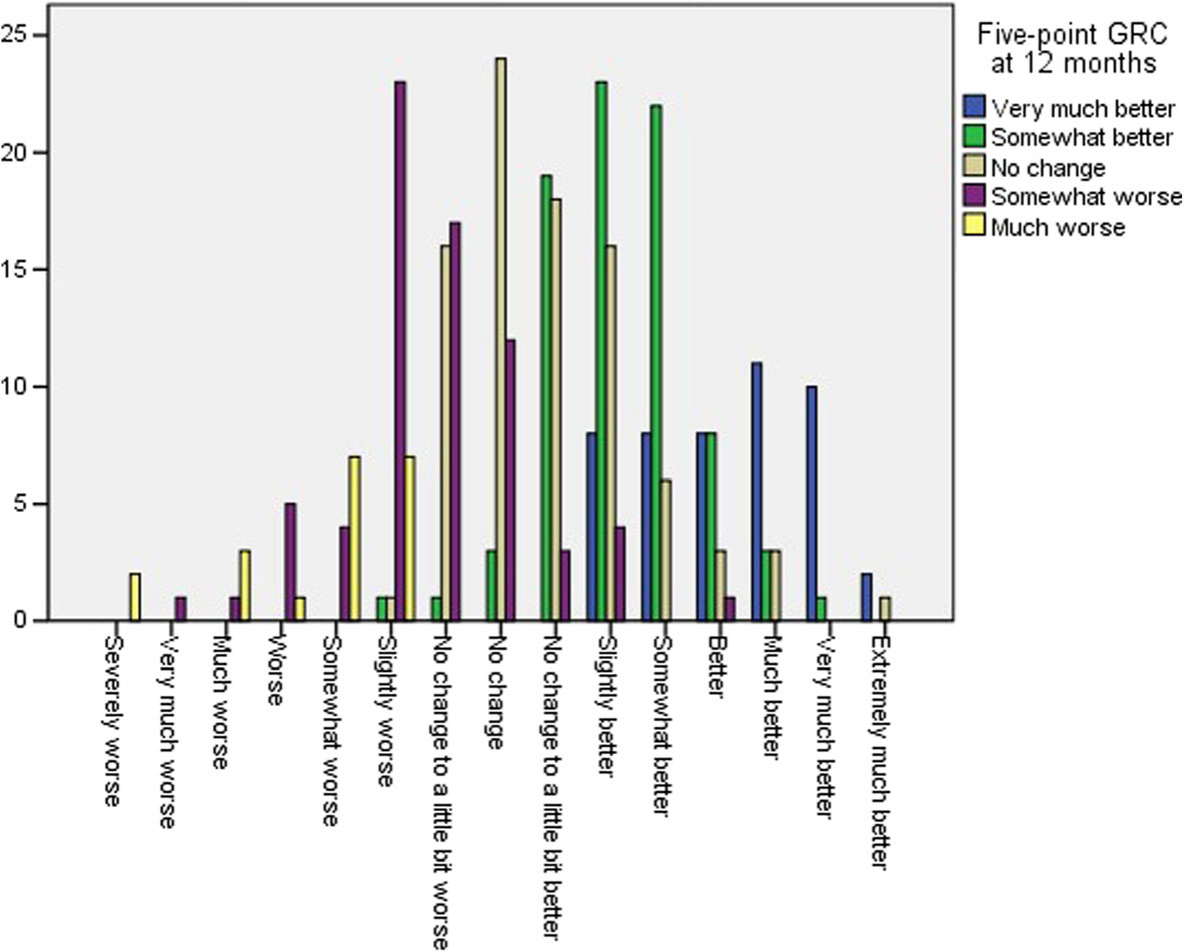
Correspondence between the five- and 15-point Global Rating of Change scale at 12 months follow-up

All change scores and 15-point anchor question scores were normally distributed. The five-point GRC at 12 months was treated as non-parametric data. At T1, one patient had a missing GRC score. No other GRC scores were missing for T2-T5. Correlations between the five-/15-point anchor questions and the health status change scores on the CAT, CCQ and SGRQ were all ≥0.30, except for CCQ and CAT at T1 (Table 3). The Spearman correlation coefficient between the five- and 15-point GRC at 12 months was 0.81.The overlap between the five-point GRC and 15-point GRC classification at 12-months was 55% based upon a cross-tabulation (Fig. 2).

**Table 3:** Correlations between health status change scores and the Global Rating of Change anchor questions

	15-point GRC T1	15-point GRC T2	15-point GRC T3	15-point GRC T4	15-point GRC T5	Five-point GRC T5
N of Patients	451	355	319	304	309	309
CAT change score	− 0.23	− 0.33	− 0.40	− 0.43	− 0.41	0.46
CCQ total change score	−0.29	− 0.42	− 0.44	−0.48	− 0.47	**0.50**
SGRQ total change score	−0.30	−0.48	**− 0.51**	**−0.58**	**− 0.54**	**0.57**

Data reported as Pearson or Spearman correlation coefficients between the health status change scores and the anchor questions. Correlations ≥0.50 are highlighted bold

*CAT* COPD Assessment Test, *CCQ* Clinical COPD Questionnaire, *GRC* Global Rating of Change scale, *SGRQ* St. George’s Respiratory Questionnaire, *T1* Measurement at discharge, *T2* 3 months follow-up, *T3* 6 months follow-up, *T4* 9 months follow-up, *T5* 12 months follow-up

A subset of the total patient population, indicated a minimal improvement according to their GRC score. Patients indicating *a minimal improvement* on the *15-point GRC* (scores of + 2 or + 3) noted significant absolute mean changes between the start of pulmonary rehabilitation and twelve months follow-up measurement of − 2.8 (95% CI −4.2 to − 1.4) on the CAT; − 0.5 (95% CI −0.7 to − 0.3) on the CCQ; and − 8.8 (95% CI −11.8 to − 5.8) on the SGRQ (Table 4). MCID estimates ranged from − 3.1 to − 2.3 for CAT; − 0.6 to − 0.4 for CCQ; and from − 10.3 to − 7.6 for the SGRQ. Mean change scores of those patients feeling *somewhat better* on the *five-point GRC* after 12 months were − 1.4 for CAT (95% CI -2.7 to − 0.1), − 0.3 for CCQ (95% CI −0.5 to − 0.2), and − 7.7 for SGRQ (95% CI −10.5 to − 4.8) (Table 4).

**Table 4:** MCID estimates for minimally improved patients as indicated on the GRC during follow-up

Measurement period	N	CAT	CAT Standardized	CCQ Total	CCQ Total Standardized	SGRQ Total
15-point GRC T1-T0	196	−3.1*±5.3 (− 3.9 to − 2.4)	− 7.8*±13.2 (− 9.7 to − 5.9)	−0.6*±0.8 (− 0.7 to − 0.4)	− 9.3*±14.0 (− 11.3 to − 7.3)	− 8.4*±11.8 (− 10.1 to − 6.7)
15-point GRC T2-T0	107	−2.7*±6.4 (− 4.0 to − 1.5)	−6.9*±16.1 (− 9.9 to − 3.8)	−0.4*±1.0 (− 0.6 to − 0.2)	− 7.3*±16.8 (− 10.5 to − 4.0)	− 7.6*±13.8 (− 10.2 to − 4.9)
15-point GRC T3-T0	96	− 2.7*±6.7 (− 4.1 to − 1.4)	−6.8*±16.7 (− 10.2 to − 3.5)	−0.4*±1.1 (− 0.6 to − 0.2)	− 7.0*±18.0 (− 10.6 to − 3.3)	− 9.2*±14.0 (− 12.1 to − 6.3)
15-point GRC T4-T0	80	−2.3*±6.1 (− 3.7 to − 1.0)	−5.8*±15.2 (− 9.2 to − 2.4)	−0.5*±0.8 (− 0.7 to − 0.3)	− 7.6*±13.8 (− 10.8 to − 4.7)	− 10.3*±12.9 (− 13.2 to − 7.4)
15-point GRC T5-T0	88	−2.8*±6.7 (− 4.2 to − 1.4)	−7.0*±16.7 (− 10.5 to − 3.5)	− 0.5*±1.0 (− 0.7 to − 0.3)	− 8.3*±16.3 (− 11.7 to − 4.8)	− 8.8*±14.1 (− 11.8 to − 5.8)
Five-point GRC T5-T0	81	−1.4*±5.9 (− 2.7 to − 0.1)	−3.5*±14.7 (− 6.7 to − 0.3)	−0.3*±0.8 (− 0.5 to − 0.2)	− 5.5*±13.8 (− 8.7 to − 2.5)	−7.7*±12.9 (− 10.5 to − 4.8)

Minimal change scores reported as mean ± SD (95%CI). Change is measured compared to baseline. Negative values represent improvement for CAT, CCQ and SGRQ. CAT and CCQ were standardized into a scale from zero to 100 to be comparable with SGRQ

*Significance of change scores *p* < 0.05 at T1/T2/T3/T4/T5 compared to baseline (T0)

*95%CI* 95% Confidence Interval, *CAT* COPD Assessment Test, *CCQ* Clinical COPD Questionnaire, *GRC* Global Rating of Change scale, *N* number of patients, *SD* Standard Deviation, *SGRQ* St. George’s Respiratory Questionnaire, *T0* Measurement at baseline, *T1* Measurement at discharge, *T2* 3 months follow-up, *T3* 6 months follow-up, *T4* 9 months follow-up, *T5* 12 months follow-up

### Tests of significance between MCID estimates

ICC values ranged 0.5–0.7 for CAT, 0.5–0.7 for CCQ, and 0.6–0.7 for SGRQ (Table 5).

**Table 5:** Determination of appropriate Confidence Intervals (CI) testing for significantly different MCIDs between time points

	15-point GRC T0-T1	15-point GRC T0-T2	15-point GRC T0-T3	15-point GRC T0-T4	15-point GRC T0-T5	Five-point GRC T0-T5
CAT						
Mean change	−3.1	−2.7	− 2.7	− 2.3	−2.8	−1.4
SD of the change	5.3	6.4	6.7	6.1	6.7	5.9
N of patients	196	107	96	80	88	81
ICC	0.7	0.6	0.5	0.6	0.5	0.6
Z score required	0.62	0.98	0.98	0.98	0.98	0.98
Standard Error	0.4	0.6	0.7	0.7	0.7	0.7
CI	−3.4 to −2.9	−3.4 to −2.1	−3.4 to −2.1	−3.0 to −1.6	−3.5 to − 2.1	−2.0 to − 0.8
Standardized mean change and CI	−7.8 (− 8.4 to − 7.2)	−6.9 (− 8.4 to −5.3)	−6.8 (−8.5 to −5.2)	−5.8 (− 7.4 to −4.1)	− 7.0 (− 8.8 to − 5.3)	− 3.5 (− 5.1 to − 1.90)
CCQ Total						
Mean change	−0.6	− 0.4	− 0.4	− 0.5	−0.5	− 0.3
SD of the change	0.8	1.0	1.1	0.8	1.0	0.8
N of patients	196	107	96	80	88	81
ICC	0.7	0.6	0.5	0.7	0.5	0.6
Z score required	0.62	0.98	0.98	0.62	0.98	0.98
Standard Error	0.1	0.1	0.1	0.1	0.1	0.1
CI	−0.7 to −0.5	−0.5 to −0.3	−0.5 to −0.3	−0.6 to − 0.4	−0.6 to − 0.4	−0.4 to 0.2
Standardized mean change and CI	−9.3 (−10.0 to −8.7)	−7.3 (−9.0 to −5.7)	−7.0 (− 8.8 to −5.2)	−7.7 (− 8.7 to −6.7)	− 8.3 (− 10.0 to − 6.7)	− 5.5 (− 7.0 to −4.0)
SGRQ Total						
Mean change	−8.4	−7.6	− 9.2	− 10.3	−8.8	− 7.7
SD of the change	11.8	13.8	14.0	12.9	14.1	12.9
N of patients	196	107	96	80	88	81
ICC	0.7	0.7	0.7	0.7	0.6	0.6
Z score required	0.62	0.62	0.62	0.62	0.98	0.98
Standard Error	0.8	1.3	1.4	1.4	1.5	1.4
CI	−8.9 to −7.9	−8.4 to −6.8	−10.1 to −8.3	−11.2 to −9.4	−10.3 to − 7.4	−9.1 to − 6.3

*CAT* COPD Assessment Test, *CCQ* Clinical COPD Questionnaire, *CI* Confidence Interval, *GRC* Global Rating of Change scale, *ICC* Intraclass Correlation Coefficient, *N* Number of Patients, *SD* Standard Deviation, *SGRQ* St. George’s Respiratory Questionnaire, *T0* Measurement at baseline, *T1* Measurement at discharge, *T2* 3 months follow-up, *T3* 6 months follow-up, *T4* 9 months follow-up, *T5* 12 months follow-up

**Fig. 3:**
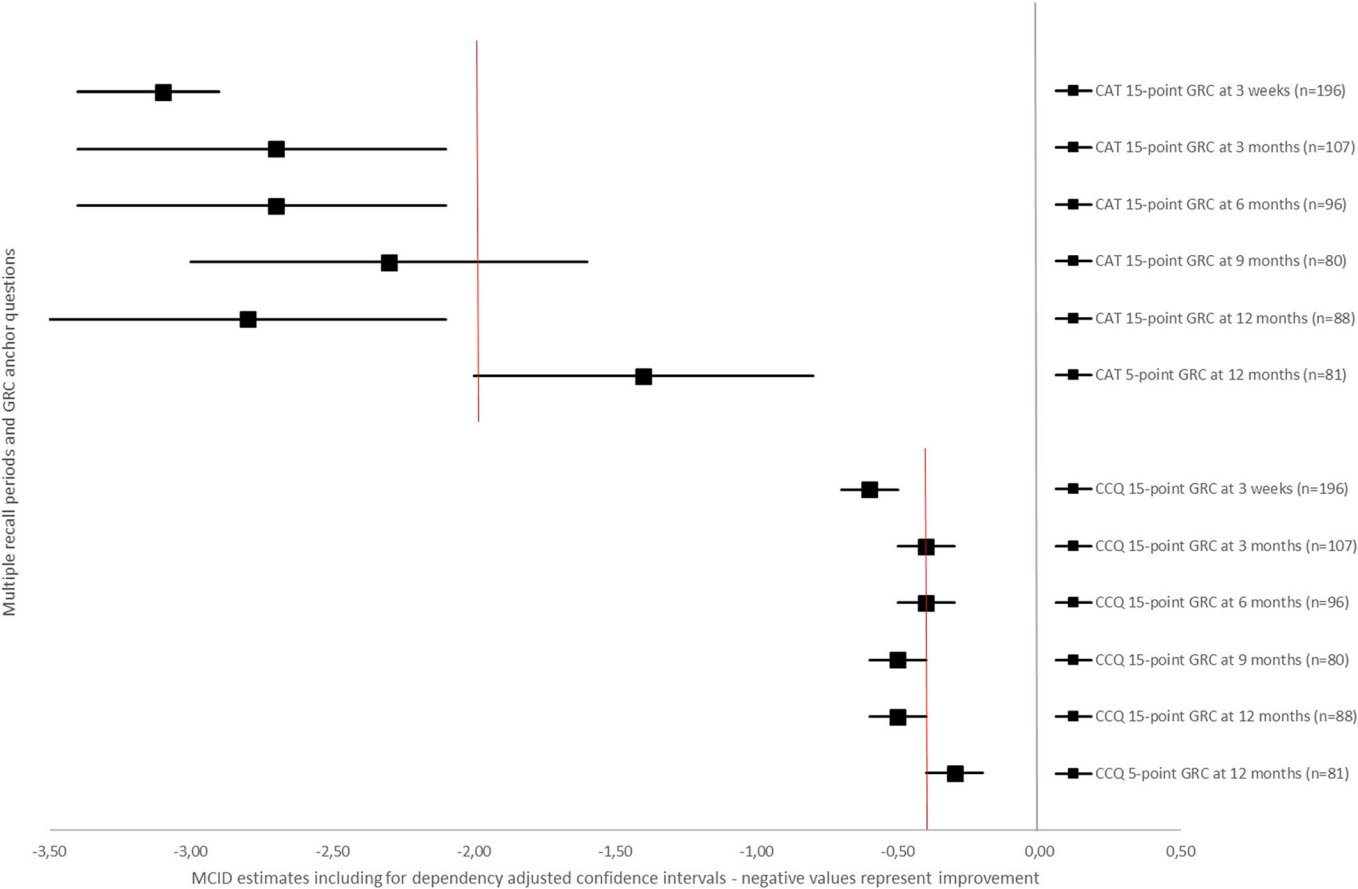
MCID estimates with for dependency adjusted confidence intervals for CAT and CCQ total score. Data are presented as MCID estimates (squares) and their respective confidence interval (horizontal line) adjusted for the dependency of the data. The red vertical lines represents the MCID estimates for CAT and CCQ total score obtained from the literature. Negative values represent improvement in health status

**Fig. 4:**
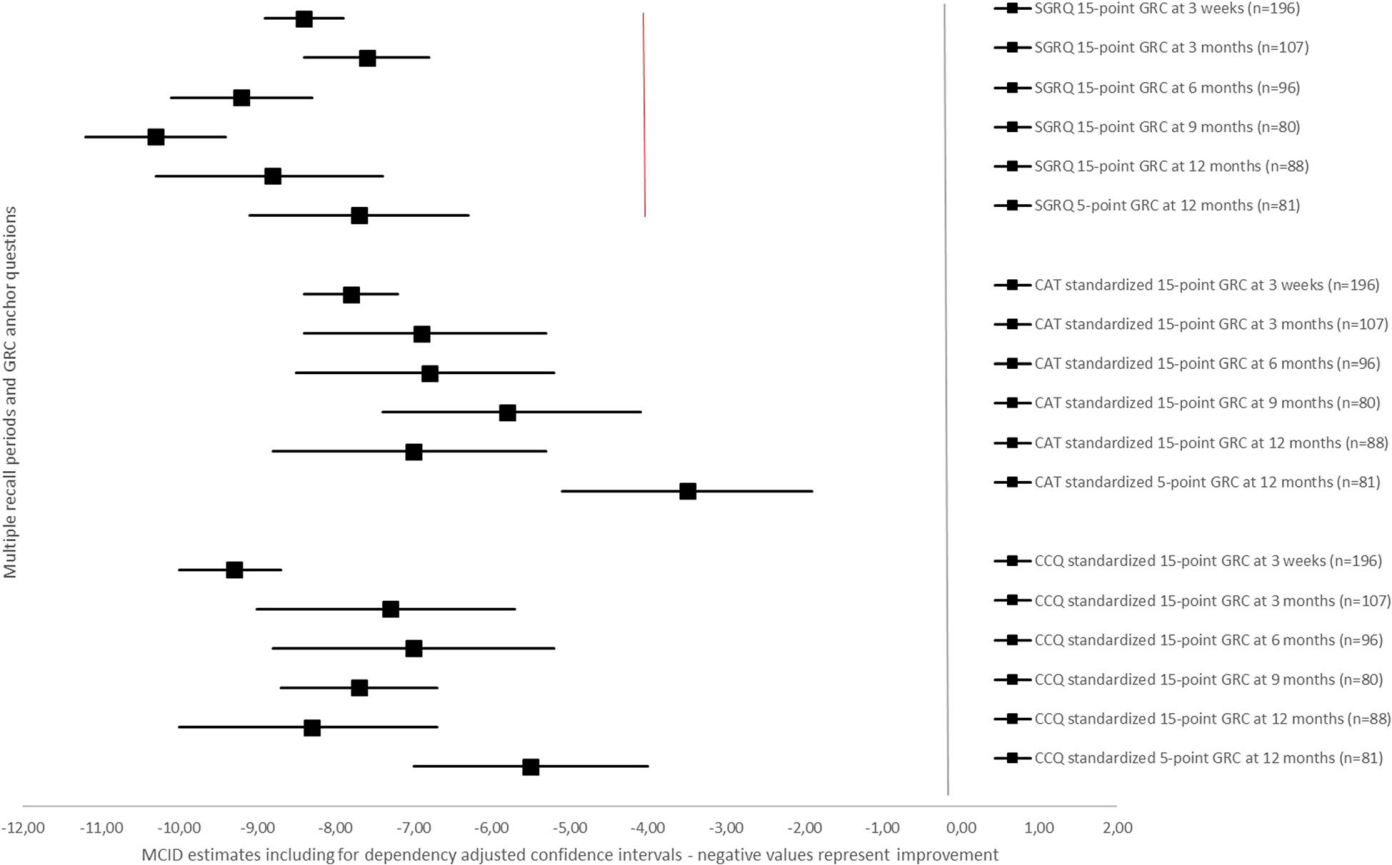
MCID estimates with for dependency adjusted confidence intervals for SGRQ total score including standardized estimates for CAT and CCQ total scores. Data are presented as MCID estimates (squares) and their respective confidence interval (horizontal line) adjusted for the dependency of the data. The red vertical line represents the MCID estimate for SGRQ total score obtained from the literature. Negative values represent improvement in health status

Figures 3 and 4 visually plot the MCID estimates for CAT, CCQ and SGRQ including their respective adjusted confidence intervals for each recall period on both GRCs. Overlap was present for all CAT MCID estimates, except for the twelve months estimate using the five-point anchor question compared with the 15-point GRC. A significantly absolute lower MCID estimate was observed for CAT using the 5-point GRC (difference −1.4: adjusted CI −2.3 to − 0.5). The MCID measured with the 15-point GRC over the nine months period as well as the MCID using the five-point anchor question overlapped with the CAT estimate from the literature of two points.

The MCID plotted for the CCQ visualized that all estimates with their corresponding CIs overlapped one another, except for the twelve months estimate with the five-point GRC compared with the 15-point GRC (Fig. 3). A significantly absolute lower MCID estimate was observed for CCQ using the 5-point GRC at 12 months (difference − 0.2: CI −0.3 to −0.1). All estimates included the MCID from the literature of 0.40 points, except for the three weeks 15-point GRC anchor question estimate.

The plot for the MCID of the SGRQ showed all ranges overlapping one another, except for the nine months 15-point GRC anchor question method, which was significantly different from the three weeks 15-point GRC estimate and three months 15-point GRC estimate (Fig. 4). There were no significant differences between the five-point and 15-point GRC at 12 months. All estimates were significantly different from the four points estimate in the literature.

## Discussion

### Summary of main findings

This study found no systematic significant differences between various recall periods of a 15-point anchor question on the MCID for improvement of the COPD health status tools CAT, CCQ and SGRQ in a PR setting. Using this 15-point GRC, MCID estimates for improvement ranged − 3.1 to − 2.3 for CAT; − 0.6 to − 0.4 for CCQ; and − 10.3 to − 7.6 for SGRQ. Higher absolute MCID estimates were observed for CAT and CCQ with a shorter three weeks recall period directly after PR, although not significant. The nine months recall period on the 15-point GRC for the SGRQ was significantly higher in absolute value when comparing with the estimates at three weeks and three months. However, an anchor question with only five answering options did result in significantly absolute lower MCIDs for CAT and CCQ in comparison with the 15-point GRC at 12 months. Estimates were − 1.4 for CAT (significant difference − 1.4), − 0.3 for CCQ (significant difference − 0.2), and − 7.7 for the SGRQ (non-significant difference − 1.1).

### Interpretation of findings

The MCID ranges found in the current study for both CAT and CCQ were in correspondence with those available in the literature [21–28]. Recall periods on the anchor question of two to three days, three weeks and eight weeks have been used before for CAT and CCQ [21, 23, 25, 27, 28]. Most MCID estimates for the CAT in the current study were significantly higher than the two points threshold, which had been advocated using a five point GRC scale [27, 28]. Since CAT only allows for integer scores, a cut-off MCID of three points would be suggested here. For the CCQ, all recall periods and anchor question types included the 0.40 points MCID as reported in the literature, although our estimates were closer to 0.50 points [21–26]. Both five-point and 15-point GRCs were used generating a 0.40 MCID estimate for the CCQ [21, 23]. The estimates for the SGRQ in the current study were significantly higher compared with the existing four points MCID, which is used extensively in scientific research [29, 31]. This MCID was among others based upon a five-point question requiring COPD patients to assess the treatment effects over a 16-week period. It did not require patients to assess their experienced change in health status, hence may result in a different MCID. The current study provided additional support to the recommendation by Welling et al. [30] and Alma et al. [25] that the MCID of the SGRQ of four points should be set higher.

There was a remarkable significant difference between the five-point and 15-point anchor question scale in estimating the MCIDs for CAT and CCQ at 12 months, although the Spearman correlation between both anchor scales was strong. However, the classification of patients according to both GRCs was only for 55% consistent, resulting thus in a different categorization of the degree of change assessed by patients themselves. Although the 15-point GRC was analysed as a seven-point scale, the patients had 15 answering options to choose from, compared with five on the other GRC. Too few reply options on an anchor question might lead to loss of relevant information, leading to less discriminative power and lower sensitivity [9]. It may result into lower MCIDs. This seems to be the case for the current study for both CAT and CCQ, and to a lesser extent for SGRQ as well. Earlier studies used only five-point GRCs for CAT and SGRQ [27–29, 31]. These studies showed lower absolute MCIDs. A five-point anchor scale may therefore not discriminate sufficiently. Kamper S.J et al. recommended to include seven to 11 reply options for optimal discrimination [9]. Another difference between the current five-point and 15-point GRC was that the first one was a verbal scale, while the latter one was a numeric scale. Possibly this has influenced the classification as words may result in a different perception in comparison to numbers.

Using an anchor question to determine an instrument’s MCID is common practice [6–8]. Jaeschke et al. were the first to use this approach in determining the MCID of the Chronic Respiratory Questionnaire (CRQ) using a 15-point Likert scale GRC [5]. Since then many have adopted this method, but have also applied alternative versions to determine the MCID. The approach is easy to administer and the single best measure of the significance of change from the patient’s perspective [9–11]. However, anchor questions rely on the patient’s ability to recall their former health state [9–11]. Accurate recall is determined by factors such as forgetting, more recent (impactful) health events, and current mood state [11]. Global Rating of Change scales may therefore not provide an accurate reflection of the real experienced change due to these recall biases.

It has been speculated that longer recall periods would lead to less accurate estimates of change and even to different MCIDs [10, 38–42]. Evaluation of change turned out to be more correlated with the current health state and severity of experienced symptoms, rather than with the former (baseline) condition [9, 10, 41–48]. There are, however, also studies that did not find specific differences between recall periods [39, 49–51]. There is no single optimal recall period [39, 51]. The required window is dependent upon whether or not acute effects need to be measured, whether acute events occur, as well as the nature of the disease [39, 52]. Longer recall periods may therefore be appropriate for chronic conditions with slow changes. It was argued that the optimal length for measuring change on a PRO in COPD would be six to 12 months [53]. A recall period of more than one year could lead to problems due to the progressive nature of the disease. In addition to the impact of recall bias, a patient’s evaluation of a specific health state might change over time due to a response shift [54]. This concept refers to a change in the meaning of the concept HRQoL for the patient. Response shift was demonstrated to have an influence on the MCID in HRQoL tools in breast cancer research [55]. Evidence for the influence of response shift as well as recall bias on the MCID of COPD health status is currently absent in the literature.

The current study had a fixed recall moment, which was related to the start of an intense PR program. The effects of PR would be expected to remain over a longer period of time, leading to less exacerbations and less acute changes in the health state of the COPD patients [56]. Jones et al. [53] recommended measurement of PROs in COPD over a 6–12 months period as the optimal recall period, which our study did. The assessment of change compared with the start of PR, the expected stability of COPD symptoms over time after PR and the use of the optimal recall period might help explain why this study found stable MCID estimates during follow-up.

Correlations between the anchor question and the health status change scores were sufficient to be used as anchor, except for the three week measurement period. It may, therefore, not be surprising that those estimates were especially for CAT and CCQ higher than the other MCID estimates. Evaluating change directly after an impacting event, such as PR or exacerbations, could potentially bias the MCID measurement of an instrument. The estimates of the SGRQ seemed rather stable over time, perhaps because SGRQ is a more extensive and lengthier tool in comparison to the CAT and CCQ.

### Strengths and limitations

This is the first study to investigate the impact of the recall period of the patient’s GRC and its design on the MCID for improvement of COPD health status tools. It is to the best of our knowledge the only study, which measured the MCID of CAT, CCQ and SGRQ in one study over multiple study periods, and included a unique test of significance for the MCID according to the methods of Afshartous et al. [37]. In the current study, MCIDs were tested over multiple periods of time. No correction for multiple testing was made, risking an increase in the probability to run a type I error. However, since this was a diagnostic study, we considered this to be of limited importance as there is no intention to make a formal statement about efficacy or safety based on hypothesis testing [57]. Furthermore, the confidence intervals for the MCID estimates were adjusted for the dependency of multiple follow-up data.

The results found in this study are valid for a PR setting. As MCIDs may differ per setting, the results need not necessarily be valid in other populations [11]. However, our results were in line with the existing MCIDs in the literature, which were also determined outside the field of PR. MCIDs were determined based upon a patient’s perspective of their health status change. No clinician, neither the patient, was involved to make a judgement about the *clinical* relevance of the perceived change though. Correlations between the GRCs and the health status questionnaires were sufficient according to pre-determined criteria, however in fact these correlations are still only small to moderate.

Another limitation is that the data used in this study were based on improvement only, as the number of patients deteriorating for each follow-up period was small to allow for significance testing. MCIDs for improvement may, however, differ from those for deterioration [11]. Furthermore, this study determined the MCID over different recall periods using the 15-point GRC scale. The five-point anchor question was, however, only measured over a twelve month period. It would not be possible to conclude whether recall bias occurred for a five-point GRC. Last, the anchor-based MCID technique can be considered a population-based figure, rather than a reflection of the individual’s change [6–8, 11]. This is a limitation of the technique in itself. Using a larger sample would lead to regression to the mean of the MCID estimate, which is less subject to larger changes in an individual’s health state.

### Implications for future clinical practice and theory

No other evidence exists for the impact of the recall period and the design of the anchor question on the determination of MCIDs in COPD health status. Ideally, more research is needed to confirm or falsify the current findings in both a PR and other settings. It would be recommended to use multiple patient-referencing anchors over multiple periods of time to carefully estimate an instrument’s MCID. Multiple MCIDs might potentially apply to practice for different time periods of measurement used in clinical trials. However, this study was the first to suggest otherwise. It indicated a differentiation might be needed between measurement of change directly after an impacting event and in stable patients, as this may be an important factor influencing recall bias.

## Conclusions

Various recall periods on a 15-point anchor question seemed not to be associated with systematic significant differences in the MCIDs for improvement of the CAT, CCQ and SGRQ, with the exception of the shortest 3-week measurement period directly after PR for CAT and CCQ, which led to absolute higher MCID estimates. Measuring change with a shorter recall period directly after an impacting event might potentially bias measurement. Using an anchor question with less answering options over a one-year period of time in determining an instrument’s MCID may also coincide with (significantly) lower absolute MCID estimates as less discriminative options might be available for the patient.

## Additional file

Additional file 1: **Figure S1.** 15-point Global Rating of Change anchor question used at each follow-up moment. **Figure S2.** Five-point Global Rating of Change anchor question used at 12-months follow-up. (DOCX 163 kb)

## Abbreviations

CAT: COPD Assessment Test
CCQ: Clinical COPD Questionnaire
CI: Confidence Interval
COPD: Chronic Obstructive Pulmonary Disease
CRQ: Chronic Respiratory Questionnaire
FEV1%Pred: Forced Expiratory Volume in 1 s % predicted
GOLD: Global initiative for Obstructive Lung Diseases
GRC: Global Rating of Change scale
HRQoL: Health-Related Quality of Life
ICC: Intraclass Correlation Coefficient
MCID: Minimal Clinically Important Difference
N: Number of patients
PR: Pulmonary Rehabilitation
PRO: Patient-Reported Outcome
RIMTCORE: Routine Inspiratory Muscle Training within COPD Rehabilitation
SD: Standard Deviation
SF-36: Short-Form 36
SGRQ: St. George's Respiratory Questionnaire
T0: Pre-rehabilitation measurement
T1: Post-rehabilitation measurement
T2: Time-point 2: 3 month follow-up
T3: Time-point 3: 6 month follow-up
T4: Time-point 4: 9 month follow-up
T5: Time-point 5: 12 month follow-up
