# Supplementary material for: Assessing health status over time: impact of recall period and anchor question on the minimal clinically important difference of copd health status tools
Source: Health Qual Life Outcomes. 2018 Jun 26;16:130. doi: 10.1186/s12955-018-0950-7 (PMC6019834; doi:10.1186/s12955-018-0950-7)
Supplement: Supplementary file 1 — Figure S1. 15-point Global Rating of Change anchor question used at each follow-up moment. Figure S2. Five-point Global Rating of Change anchor question used at 12-months follow-up. (DOCX 163 kb) [file 12955_2018_950_MOESM1_ESM.docx]

**Supplementary Figure 1: 15-point Global Rating of Change anchor question used at each follow-up moment**

**Supplementary Figure 2: Five-point Global Rating of Change anchor question used at 12-months follow-up**

|  | Derzeit viel besser | Derzeit etwas besser | Etwa wie vor einem Jahr | Derzeit etwas schlechter | Derzeit viel schlechter |
| --- | --- | --- | --- | --- | --- |
| Im Vergleich zum *vervangenen Jahr*, wie würden Sie Ihren derzeitigen Gesundheitszustand beschreiben? | 1 | 2 | 3 | 4 | 5 |
